# Thin Amelanotic and Hypomelanotic Melanoma: Clinicopathological and Dermoscopic Features

**DOI:** 10.3390/medicina60081239

**Published:** 2024-07-30

**Authors:** Giovanni Paolino, Riccardo Pampena, Sofia Maria Di Ciaccio, Andrea Carugno, Carmen Cantisani, Matteo Riccardo Di Nicola, Luigi Losco, Giulio Bortone, Santo Raffaele Mercuri, Antonio Costanzo, Marco Ardigò, Mario Valenti

**Affiliations:** 1 Unit of Dermatology and Cosmetology, IRCCS San Raffaele Scientific Institute, 20132 Milan, Italy; paolino.giovanni@hsr.it (G.P.); mercuri.santoraffaele@hsr.it (S.R.M.); 2 La Sapienza University of Rome, 00185 Rome, Italy; riccardopampena@gmail.com (R.P.); sofiamariadiciaccio@gmail.com (S.M.D.C.); 3 Department of Medicine and Surgery, University of Insubria, 21100 Varese, Italy; 4 Dermatologic Clinic, La Sapienza University of Rome, 00185 Rome, Italy; carmencantisanister@gmail.com (C.C.); giuliobortone93@gmail.com (G.B.); 5 Plastic Surgery Unit, Department of Medicine, Surgery and Dentistry, University of Salerno, 84084 Baronissi, Italy; luigi.losco@gmail.com; 6 UniSr Vita-Salute San Raffaele University, 20132 Milano, Italy; 7 Dermatology Unit, IRCCS Humanitas Research Hospital, 20089 Rozzano, Italy; antonio.costanzo@hunimed.eu (A.C.); ardigo.marco@gmail.com (M.A.); valenti.mario.92@gmail.com (M.V.)

**Keywords:** amelanotic melanoma, hypomelanotic melanoma, Breslow thickness, pheomelanin, multiple primary melanomas

## Abstract

*Background and Objectives*: Amelanotic/hypomelanotic melanomas (AHMs) account for 2–8% of all cutaneous melanomas. Due to their clinical appearance and the lack of specific dermoscopic indicators, AHMs are challenging to diagnose, particularly in thinner cutaneous lesions. The aim of our study was to evaluate the clinicopathological and dermoscopic features of thin AHMs. Identifying the baseline clinical–pathological features and dermoscopic aspects of thin AHMs is crucial to better understand this entity. *Materials and Methods*: We divided the AHM cohort into two groups based on Breslow thickness: thin (≤1.00 mm) and thick (>1.00 mm). This stratification helped identify any significant clinicopathological differences between the groups. For dermoscopic analysis, we employed the “pattern analysis” approach, which involves a simultaneous and subjective assessment of different criteria. *Results*: Out of the 2.800 melanomas analyzed for Breslow thickness, 153 were identified as AHMs. Among these, 65 patients presented with thin AHMs and 88 with thick AHMs. Red hair color and phototype II were more prevalent in patients with thin AHMs. The trunk was the most common anatomic site for thin AHMs. Patients with thin AHMs showed a higher number of multiple melanomas. Dermoscopic analysis revealed no significant difference between thin AHMs and thick AHMs, except for a more frequent occurrence of residual reticulum in thin AHMs. *Conclusions*: Thin AHMs typically affect individuals with lower phototypes and red hair color. These aspects can be related to the higher presence of pheomelanin, which provides limited protection against sun damage. This also correlates with the fact that the trunk, a site commonly exposed to intermittent sun exposure, is the primary anatomical location for thin AHMs. Multiple primary melanomas are more common in patients with thin AHMs, likely due to an intrinsic predisposition as well as greater periodic dermatologic follow-ups in this class of patients. Apart from the presence of residual reticulum, no other significant dermoscopic differences were observed, complicating the differential diagnosis between thin and thick AHMs based on dermoscopy alone.

## 1. Introduction

Amelanotic/hypomelanotic melanomas (AHMs) are melanomas characterized by less than 25% of the lesion’s surface displaying pigmentation and account for 2–8% of all cutaneous melanomas [1,2,3]. Within this spectrum, thin AHMs present greater diagnostic challenges due to their clinical appearance and absence of specific dermoscopic features. Indeed, dermoscopic algorithms for “pink tumors” significantly lack the diagnostic accuracy seen in those for pigmented lesions [4]. Consequently, identifying thin AHMs is crucial for reducing melanoma-related mortality [2,5,6].

AHM is associated with specific phenotypic and genotypic profiles, which play a pivotal role in identifying populations at risk [5,7]. Studies indicate that the phenotypic traits associated with AHM include red or blonde hair, skin phototype I, freckling, and the presence of actinic keratoses [7,8,9]. However, to our knowledge, the current literature lacks articles about the diagnosis and clinic–pathologic features of thin AHMs. There are some reports on amelanotic flat lesions, yet these lack a specific subdivision according to Breslow thickness [10,11].

Approximately 70% of new melanoma cases are classified as thin melanomas (TMs), defined by a Breslow thickness of less than 1 mm [10,11]. In the absence of other risk factors, such as ulceration, TMs exhibit a 10-year survival rate of 96% [10]. However, for amelanotic and/or hypochromic lesions, this survival rate may be reduced due to delays in diagnosis. Thus, in daily clinical practice, early diagnosis is paramount to reduce both the morbidity and mortality associated with melanoma. Recently, even in cases of advanced and metastatic disease, there have been significant advancements regarding the treatment of metastatic melanoma, such as target therapies (using BRAF and MEK inhibitors) or immune checkpoint inhibitors, such as PD-1, CTLA-4, or LAG-3 inhibitors [11,12,13,14,15,16,17,18,19,20,21,22,23,24].

The aim of this study was the evaluation of the clinicopathological and dermoscopic features of thin AHMs since previous studies did not differentiate between AHMs based on Breslow thickness (≤1.00 mm Breslow thickness or >1.0 mm Breslow thickness).

## 2. Materials and Methods

This is a multicenter study, including patients from IRCCS Ospedale San Raffaele (Milan, Italy), Sapienza University of Rome (Rome, Italy), and Humanitas Research Hospital (Milan, Italy). This retrospective study was performed according to the Declaration of Helsinki, which sets forth ethical principles. Given the retrospective nature of the study and the absence of invasive measures on patients or activities outside daily clinical practice, approval by the ethics committee was deemed unnecessary. Inclusion criteria for patients were age ≥ 18 and clinical/dermoscopic diagnosis of AHM with histological confirmation. We excluded patients under the age of 18, as AHMs account for 70% of pediatric melanoma cases [8]. Hypomelanotic melanomas (HMs) are defined as melanomas showing pigmentation on less than 25% of the lesion’s surface with the histological presence of focal pigmentation, while amelanotic melanomas (AMs) are defined as melanomas with clinical/dermoscopic and histological absence of pigmentation [8]. However, in this study, AMs and HMs have been evaluated together in a single sample termed AHMs.

The clinicopathological data obtained from the electronic databases of each Institute were as follows: gender, hair color, eye color, phototype, history of melanoma, anatomical site, presence of pigmentation in the lesion, histological subtype, Breslow thickness (≤1.0 mm; >1.0 mm), and presence or absence of ulceration and histologic regression.

Dermoscopic images were obtained using digital/manual dermatoscopes. We employed the “pattern analysis” approach for dermoscopic evaluation based on the simultaneous and subjective assessment of various criteria [25]. The dermoscopic criteria of interest included pigmentation, network, globules, dots, white structureless zone, white lines, and vascular patterns. The vascular patterns were classified as follows: vascular dots, vascular clods, and linear (straight, looped, curved, serpentine, helical, coiled) and arborizing vessels.

### Statistical Analysis

Assuming that the effects of the predictor variables are constant over time, we utilized Fisher’s exact test to analyze the distribution of categorical variables in a small sample and Spearman’s rho coefficient sizes to assess non-parametric rank correlations in AHMs. Subsequently, the independent predictive factors were assessed by multiple logistic regression. A *p*-value ≤ 0.05 was considered statistically significant.

## 3. Results

### 3.1. AHM Clinicopathological and Dermoscopic Features

Out of 2.800 melanomas assessed for Breslow thickness, a total of 153 (all Caucasian patients) were diagnosed as AHMs, representing 5.5% of cases. Among these, 98 patients (64% of AHM cases; N = 98) showed multiple melanomas, with an average number of 1.9 ± 2.6 melanomas per patient. All patients included in this study were outpatients who were evaluated, treated, and followed up at our Institutes. The general patient population consisted of 75 males and 78 females, with a median age of 59 years (IQR = 46–68). The most commonly affected anatomical site was the trunk (39.2%), followed by the lower limbs (29.4%) and upper limbs (21.0%). Regarding eye color, blue/grey was the most prevalent (65.8%), followed by green/hazel (27.6%) and brown (6.6%). The most frequent hair color was red (45.7%), followed by dark brown (22.2%). Additionally, most patients were classified as phototype II (49.4%), followed by type III and type I. Out of all AHM cases (N = 153), 66.0% (N = 101) were classified as AM and 34.0% (N = 52) as HM. The mean Breslow thickness was 1.9 mm (±2.1 SD), and the median thickness was 1.3 mm (IQR = 0.6–2.3). Ulceration was present in 26.1% of cases, while regression was observed in 24.2% of cases (Table 1).

### 3.2. Thin AHM Clinicopathological and Dermoscopic Features

Out of the 153 patients, 65 (42.5%) presented with an AHM ≤ 1.00 mm in Breslow thickness. Thin melanomas were slightly more common in female patients than in males. The predominant hair color was red (62.5%), and the most common phototype was phototype II (65.9%). Superficial spreading melanoma (75.4%) was the most common histotype, with the trunk as the main anatomic location (55.4%). Patients with thin melanoma had an average of 3 ± 4.5 melanomas. The mean Breslow thickness was 0.6 ± 0.2 mm.

**Figure 1:**
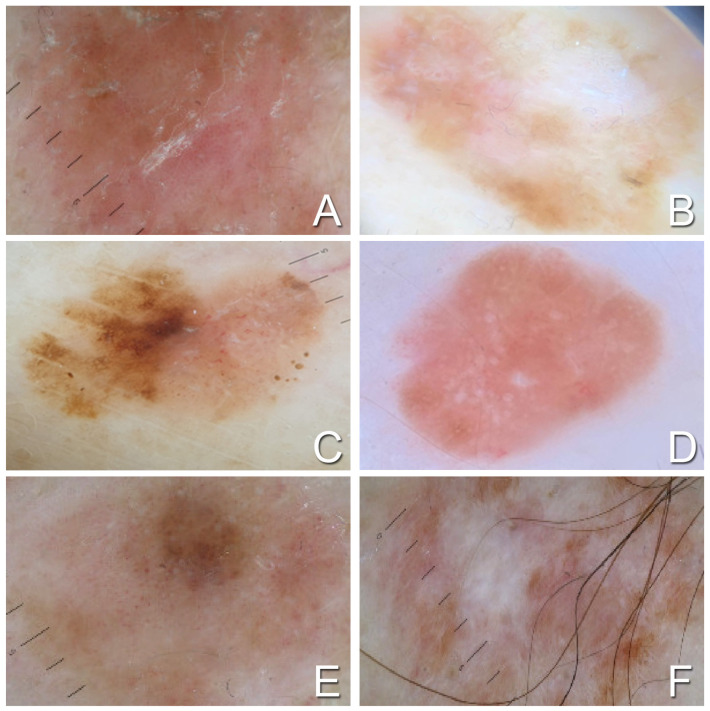
Dermoscopy features hypomelanotic and amelanotic thin melanomas. (**A**) Hypomelanotic melanoma of the trunk, 0.6 mm Breslow, in a 68-year-old man; (**B**) hypomelanotic melanoma of the back, 0.6 mm Breslow in a 39-year-old man; (**C**) hypomelanotic melanoma of the lower limb, 0.8 mm Breslow in a 72-year-old woman; (**D**) amelanotic melanoma of the lower limb, 0.5 mm Breslow in a 32-year-old woman; (**E**) hypomelanotic melanoma of the trunk, 0.5 mm Breslow, in a 66-year-old man; and (**F**) hypomelanotic melanoma of the trunk, 0.65 mm Breslow, in a 68-year-old man.

Dermoscopic analysis was available for 63 patients (Figure 1). Scar-like depigmentation was present in 45.5% of thin AHM cases. The main vascular patterns (Table 2) included linear looped, dots, and linear curved, each observed in 40.9% of cases. Dermoscopically, the reticulum was evident in 54.5% of thin AHM cases. Other clinicopathologic and dermoscopic features of thin AHMs are reported in Table 1 and Table 2.

### 3.3. Analysis of Thin AHMs (≤1 mm) vs. AHMs > 1 mm

In comparing thin and thick AHMs, we found that the dark/brown hair color was more common in AHMs > 1 mm, while red was more common in thin AHMs (*p* < 0.001). Phototype III was more frequent in AHMs > 1 mm, whereas phototype II was predominant in thin melanomas (*p* = 0.006). Nodular melanoma was more common in AHMs > 1 mm, while superficial spreading melanoma was more common in thin AHMs (*p* < 0.001). The lower limbs were the primary anatomic location for AHMs > 1 mm, while the trunk was the main site for thin AHMs (*p* < 0.001). Patients with thin AHMs showed a higher number of multiple melanomas compared to those with thick AHMs, with an average of 3 versus 1.4 (*p* = 0.006) (Table 1).

Dermoscopic analysis revealed no significant differences between thin and thick AHMs, except for the presence of residual reticulum, which was more common in thin AHMs (54.5% versus 17.1%; *p* = 0.002) (Table 2).

## 4. Discussion

The diagnosis of pink skin lesions represents one of the greatest challenges in dermatology [4,7,26,27]. Early diagnosis of AHM is crucial to limiting the mortality associated with cutaneous melanoma [7]. However, the diagnosis becomes particularly challenging when AHM is characterized by thin and flat cutaneous lesions due to limited diagnostic clues. Moreover, the main clinicopathological analysis of AHMs currently present in the literature predominantly focuses on thick AHM lesions, with very few articles addressing thin AHMs.

In the general AHM population, we did not find a difference between male and female patients, despite the relationship between gender and AHM still remaining controversial. Some studies have shown a female predominance [2,28,29], while others have shown a male predominance [8] or no significant differences [30]. According to our analysis, the median age of our population is comparable to those reported in previous studies [2,9,31].

Phototype II was confirmed as the most common phototype in our sample, as previously reported in the literature [8]. However, thin AHMs displayed a significant predominance of phototype I, confirming the data from the literature, finding AHMs more common in patients with phototype I [7,9]. Dark/brown hair was represented more in thick AHMs, while red hair was predominant in thin AHMs. The presence of dark/brown hair was confirmed by a higher predominance of dark/brown hair in the Italian population, while red hair is the main hair color usually present in patients with AHMs [5,32]. The differences in terms of phototypes and hair colors between thin and thick melanomas warrant further investigation. Most likely, low skin phototypes produce higher levels of pheomelanin, which yields poor protection against sun damage [5,33]. This aligns with the observation that AHMs typically appear in anatomical areas subjected to intermittent sun exposure, with the trunk being common in thin AHMs and limbs in thick AHMs.

Interestingly, 58% of the entire sample (thin + thick AHM) showed multiple melanomas; specifically, multiple melanomas were more common in patients with thin AHMs. This may be related to the higher number of dermatological visits undertaken by patients with thin AHMs, who may also have a higher predisposition to develop multiple melanomas [8].

Superficial spreading melanoma (SSM) was the most represented histotype in thin AHMs, while nodular melanoma (NM) was more common in thick AHMs.

Although NM is usually the most represented melanoma in patients with AHM, the presence of thin lesions in our sample confirms that AHMs are not diagnosed promptly, often leading to a late diagnosis of NM. The participation of tertiary centers specialized in dermoscopy may justify the high number of thin AHMs in our sample.

Dermoscopically, the reticulum was more common in thin AHMs. The inverse correlation between the presence of residual reticulum (as a sign of residual pigmentation) and the thickness of primary melanoma may be related to a reduction in pigmentation (hypomelanosis or amelanosis) as a result of growth-related tumor dedifferentiation [34]. As reported by other studies [1,8,35], AHM generally shows a reduced presence of ulceration. In our study, no cases of ulceration were found in thin AHMs, only in thick ones.

To date, there are few available specific dermatoscopic diagnostic criteria for AHMs. The distribution of vessels and their morphology represent the main features to assess hypopigmented and amelanotic cutaneous lesions [4,36]. Rosenthal et al. [37] proposed an assessment based initially on non-vascular parameters (also known as “white clues”, such as white structureless zones and white lines), while Menzies et al. [4] confirmed the presence of a scar-like depigmentation as a specific positive predictor in the diagnosis of AHM. In our sample, scar-like depigmentation was more common in thick melanoma than in thin melanoma (48.8% versus 45.5%), as were white lines (29.3% versus 13.6%) (Table 2). However, these results did not reach statistical significance; as well as this, we did not find any statistically significant difference between the presence of histological regression in primary thin AHMs versus thick AHMs, despite finding a higher presence of histological regression in thin AHMs (30.7%) than thick AHMs (19.3%) (Table 1). The higher presence of regression in thin AHMs may be related to residual reticulum (residual pigmentation), which can induce an immune reaction. Indeed, regression in melanoma is related to the inflammatory response against specific melanocyte-associated antigens involved in skin pigmentation [38]. In this regard, uncontrolled melanogenesis may intermediate on RNA, DNA, and regulatory proteins, generating a pro-mutagenic environment and contributing to melanomagenesis [39]. In addition, recent studies found that the non-calcemic vitamin D3 derivative (20[OH]D3) can target NF-κB, therefore regulating the induction of melanoma and its relative progression. Accordingly, vitamin D receptor (VDR), as well as CYP27B1, are more under-expressed in highly pigmented melanomas than in AHMs [11,40,41,42]; as reported in the literature, all these aspects can justify the better survival rates (disease-free survival and overall survival) observed in amelanotic melanomas compared to pigmented melanomas [39,43,44]. Therefore, the worse survival found in some studies in patients with amelanotic melanoma compared to pigmented melanoma depends on the Breslow thickness (which is greater in AHMs compared to pigmented melanomas due to delayed clinical diagnosis) rather than on AHM’s biology [8]. Indeed, the absence of pigmentation in AHMs is not associated with the poor differentiation of melanocytes, further justifying a generally worse prognosis in patients with pigmented melanoma [43]. In addition, melanoma (above all, pigmented melanoma) produces classical regulators or mediators of the hypothalamic–pituitary–adrenal (HPA) axis, which, when released into circulation or acting on nerve endings, favor tumor progression by affecting the local environment and systemic response [45,46,47,48,49,50,51,52].

Finally, the vascular pattern is of utmost importance in the diagnosis of AHMs [4,36]. In this regard, we confirmed the prevalence of linear looped and dotted vessels as the main vascular elements in AHM without any difference between thin and thick lesions.

The present work has some limitations; indeed, we acknowledge the small sample size, but as AHM is a rare malignancy, it might be hard to collect larger samples. Finally, the retrospective nature of the study may have influenced the results.

## 5. Conclusions

Diagnosing AHM remains a significant challenge in daily clinical practice, particularly when dealing with thin lesions. Identifying the baseline clinical–pathological features and dermoscopic aspects of thin AHMs is crucial in order to better understand this entity. We found that thin AHMs are mainly characterized by lower phototypes and red hair color. The trunk was the main anatomic site of thin AHMs, suggesting the important role of intermittent sun exposure. In addition, patients with thin AHMs are more likely to develop multiple primary melanomas. Histologically, superficial spreading melanoma was the main histotype detected in thin AHMs, while, regarding dermoscopy, the presence of residual reticulum was more frequent in thin AHMs than in thick AHMs. Finally, other dermatoscopic features, such as scar-like depigmentation, white lines, linear looped vessels, and dot vessels, were the main aspects detected in both thin and thick primary AHMs. Currently, dermoscopy does not aid in distinguishing between thin and thick AHMs. Further investigations are essential to enhance the diagnosis of this challenging type of melanoma.

## Figures and Tables

**Table 1:** Clinicopathologic baselines of patients.

Variables			Melanoma						*p* Value
			Thin (In Situ and <1 mm)		Thick (≥1 mm)		Total		
			N	%	N	%	N	%	
Demographics	Mean age ± SD		59.0 ± 14.2 (n:65)		55.8 ± 16.7 (n:85)		57.2 ± 15.7 (n:150)		0.224
	Gender (N:153, 65 thin, 88 thick)	M	28	43.0%	47	53.4%	75	49.0%	0.206
		F	37	57.0%	41	46.6%	78	51.0%	
Clinical features	Eye color (N:76, 38 thin, 38 thick)	1	28	73.7%	22	57.9%	50	65.8%	0.056
		2	10	26.3%	11	28.9%	21	27.6%	
		3	0	0.0%	5	13.2%	5	6.6%	
	Hair color (N:81, 40 thin, 41 thick)	1	25	62.5%	12	29.2%	37	45.7%	<0.001
		2	10	25.0%	5	12.2%	15	18.5%	
		3	3	7.5%	5	12.2%	8	9.9%	
		4	2	5.0%	16	39.0%	18	22.2%	
		5	0	0.0%	3	7.3%	3	3.7%	
	Phototype (N:89, 41 thin, 48 thick)	1	8	19.5%	6	12.5%	14	15.7%	0.006
		2	27	65.9%	17	35.4%	44	49.4%	
		3	5	12.2%	16	33.3%	21	23.6%	
		4	1	2.4%	7	14.5%	8	9.0%	
		5	0	0.0%	2	4.1%	2	2.2%	
Melanoma data	Familial history (N:153, 65 thin, 88 thick)	no	3	4.6%	9	10.2%	12	7.8%	0.05
		melanoma	4	6.1%	3	3.4%	7	4.6%	
		other tumors	3	4.6%	0	0.0%	3	2.0%	
		unknown	55	8.5%	76	86.3%	131	85.6%	
	Histotype (N:153, 65 thin, 88 thick)	LMM	0	0.0%	1	1.1%	1	0.6%	<0.001
		SSM	49	75.4%	25	28.4%	74	48.3%	
		angiomatoid	0	0.0%	1	1.1%	1	0.6%	
		desmoplastic	0	0.0%	1	1.1%	1	0.6%	
		lentiginous	2	3.0%	1	1.1%	3	2.0%	
		nodular	6	9.2%	36	41.0%	42	27.4%	
		spitzoid	2	3.0%	6	6.9%	8	5.2%	
		unknown	6	9.2%	17	19.3%	23	15.0%	
	Location (N:153, 65 thin, 88 thick)	HN	0	0.0%	16	18.2%	16	10.4%	<0.001
		trunk	36	55.4%	24	27.3%	60	39.2%	
		upper limbs	9	13.9%	23	26.1%	32	21.0%	
		lower limbs	20	30.7%	25	28.4%	45	29.4%	
	Mean N of melanomas ± SD		3.0 ± 4.5 (n:28)		1.4 ± 1.0 (n:70)		1.9 ± 2.6 (n:98)		0.006
	Clinical pigmentation (N:153, 65 thin, 88 thick)	amelanotic	38	58.4%	63	71.6%	101	66.0%	0.09
		hypomelanotic	27	41.5%	25	28.4%	52	34.0%	
Histology	Mean Breslow (mm) (in situ excluded)		0.6 ± 0.2 (n:58)		2.8 ± 2.3 (n:88)		1.9 ± 2.1 (n:146)		<0.001
	Clark (N:125, 47 thin, 78 thick)	I	17	36.2%	8	10.2%	25	20.0%	<0.001
		II	19	40.4%	5	6.4%	24	19.2%	
		III	7	14.9%	21	27.0%	28	22.4%	
		IV	1	2.1%	39	50.0%	40	32.0%	
		V	3	6.4%	5	6.4%	8	6.4%	
	Mitotic rate (N:105, 38 thin; 67 thick)	0	16	42.1%	9	13.4%	25	23.8%	<0.001
		1	8	21.0%	2	3.0%	10	9.5%	
		2	14	36.9%	56	83.6%	70	66.7%	
	Ulceration (N:153, 65 thin, 88 thick)		15	23.0%	25	28.4%	40	26.1%	0.796
	Regression (N:153, 65 thin, 88 thick)		20	30.7%	17	19.3%	37	24.2%	0.137
Total			65		88		153		

**Table 2:** Dermoscopic aspects and analysis.

Dermoscopic Criteria	Thin		Thick		Total		*p* Value
	N:22	%	N:41	%	N:63	%	
Reticulum	12	54.5%	7	17.1%	19	30.2%	0.002
Scar-Like Depigmentation	10	45.5%	20	48.8%	30	47.6%	0.926
Globules	9	40.9%	18	43.9%	27	42.9%	0.933
Dots	4	18.2%	9	22.0%	13	20.6%	0.787
White Lines (White Streaks)	3	13.6%	12	29.3%	15	23.8%	0.221
Vascular Dots	9	40.9%	20	48.8%	29	46.0%	0.78
Vascular Clods	7	31.8%	15	36.6%	22	34.9%	0.905
Vascular Linear Straight	8	36.4%	16	39.0%	24	38.1%	0.732
Vascular Linear Looped (Hairpin)	9	40.9%	26	63.4%	35	55.6%	0.27
Vascular Linear Curved (Comma)	9	40.9%	18	43.9%	27	42.9%	0.702
Vascular Linear Irregular (Serpentine)	5	22.7%	17	41.5%	22	34.9%	0.284
Vascular Linear Helical	8	36.4%	18	43.9%	26	41.3%	0.983
Vascular Linear Coiled (Glomerular)	4	18.2%	12	29.3%	16	25.4%	0.544
Arborizing Vessels	2	9.1%	6	14.6%	8	12.7%	0.64

## Data Availability

The data presented in this study are available in the main document.
